# The Impact of Headaches in Young Adults: An Analysis of Types, Triggers, and Daily Functioning Through the Headache-Attributed Restriction, Disability, Social Handicap, and Impaired Participation (HARDSHIP) Questionnaire

**DOI:** 10.7759/cureus.74792

**Published:** 2024-11-29

**Authors:** Balaji S Mahendran, Aravinthkumar Ashok Kumar, Manobharathi Manoharan

**Affiliations:** 1 Department of Community Medicine, Saveetha Medical College and Hospital, Saveetha Institute of Medical and Technical Sciences, Chennai, IND; 2 Department of Community Medicine, Krishnan, Arumugam, Periyanna (KAP) Viswanatham Government Medical College, Tiruchirappalli, IND; 3 Department of Community Medicine, Government Mohan Kumaramangalam Medical College, Salem, IND

**Keywords:** hardship, headache, headache impact, headache trigger, migraine, tension type of headache, young adults

## Abstract

Background: Headaches affect people's social, intellectual, and personal lives and are quite common worldwide, especially among young adults. Primary headaches that cause significant impairment, such as tension-type headaches (TTH) and migraines, frequently start in adolescence and early adulthood. Research on the incidence and consequences of headache problems among young people in India is scarce, especially when it comes to a variety of academic fields.

Objectives: This study aims to 1) estimate the prevalence and types of headaches among young adults aged 18-24 years and 2) identify common triggers and assess the social and academic impact of headache disorders.

Methods: Young adults from different academic backgrounds in Chennai, South India, participated in this cross-sectional survey during September and October of 2024. A total of 438 participants across the Medical, Engineering, Dental, and Arts and Science disciplines were reached via snowball sampling. The study utilized a questionnaire based on Headache-Attributed Restriction, Disability, Social Handicap, and Impaired Participation to gather data on demographics, headache types, triggers, and their impact using Google Forms. Statistical Product and Service Solutions version 21 (IBM Corp., Armonk, NY) was used for the statistical analysis, and associations were evaluated using the chi-square and analysis of variance tests.

Results: The average age of the 438 participants was 20.1 years. Of the 438 respondents, 60.9% (267 subjects) reported having headaches in the last 12 months, with 54.6% reporting headaches in the last 30 days. Among those with headache disorders (267 subjects), 35% were diagnosed with TTH, 26% with migraine, and the remaining 39% had other types of headache. Compared with TTH and other headaches, migraines were linked to greater rates of academic interference, interruption of leisure activities, and absenteeism. In addition, migraineurs reported greater levels of social avoidance (46.3%) and a substantial family history (31.6%) compared to other headache disorders. The most often stated trigger was personal stress (38.7%), which was followed by academic stress, sinusitis, and sleep difficulties.

Conclusions: Headaches affect social life, everyday activities, and academic performance in young adults. They are highly prevalent and bothersome issues. More severe impairment seems to result from migraines than from TTH. To enhance the treatment of headache problems in this group, it is imperative to raise awareness, encourage early intervention, and remove obstacles to accessing healthcare.

## Introduction

Headache disorder is one of the most common nervous system disorders with the highest prevalence globally [1]. Around 3 billion individuals (40% of the adult population worldwide) had migraine or tension-type headache (TTH) in 2021, with females aged 15-49 years being most affected [2]. According to the Global Burden of Diseases Report 2021, headaches were the third leading cause for all-cause years lived with disability (YLDs), contributing to 5.2%. There was an increase in the burden of headache disorders of 0.3% in age-standardized YLD from 2010 to 2021 [3]. Headache disorders were the second most common cause of disability-adjusted life-years in young adults and adolescents (10-24 years old) in 2019 [4].

According to WHO, headaches can be classified into two main categories: primary headaches and secondary headaches. Primary headaches, which include TTHs, migraines, and cluster headaches, are not caused by underlying medical conditions and are often recurrent. In contrast, secondary headaches result from other medical issues, such as infections, head injuries, idiopathic intracranial hypertension, or vascular disorders. Primary headaches, the most prevalent type of headache, impose a significant economic burden. These disorders can affect individuals across all age groups but typically peak in adult populations [5].

The most prevalent form of primary headaches is TTH and migraines. Migraine (International Classification of Headache Disorders, ICHD-3, G43) is a recurring disorder characterized by moderate-to-severe headaches along with reversible neurological and systemic symptoms [6]. Skin allodynia, phonophobia, photophobia, and gastrointestinal problems are the notable symptoms of migraine. Alcohol, certain foods, hormonal changes, stress, fatigue, and environmental factors (bright light or loud noise) constitute the most common triggers for migraine [7]. TTH (ICHD-3 G44), the most prevalent form of headache, encompasses pain of mild-to-moderate severity, often described as a tight ring around the head bilaterally. Adequate sleep, taking suitable medication, maintaining proper posture, and employing massages help in alleviating TTH headaches [8].

Headache disorder may result in substantial personal and social consequences, including reduced quality of life; worse performance at school, college, or work; higher medical expenses and increased out-of-pocket expenditure; and a higher chance of developing additional chronic illnesses [9]. Studies reported that students with frequent headaches reported higher levels of absenteeism and difficulties concentrating during lectures, which adversely impacted their grades and overall academic achievement [9]. Similarly, employees with chronic headaches show an increased rate of absenteeism and reduced rate of work performance, which was more pronounced in those with migraine headaches [10].

Young adults aged between 18 and 24 years are more prone to the burden of headache disorders [5,11] and represent a significant proportion of the global population undergoing a pivotal phase of development and transition. However, this population has been paid less attention, ignoring the impact of headaches on them and, in turn, their reflections on society. There are few Indian studies describing the type of headaches in young adults and their prevalence, but most are outdated and have not emphasized the impact on their social and academic lives. This study aimed to evaluate the following objectives: 1) to estimate the prevalence of headaches among young adults aged 18-24 years and the types of headaches, 2) to elucidate the triggers associated with the headaches, and 3) to determine the social and academic impact of headaches on young adults.

## Materials and methods

Study setting and population

Young adults aged 18-24 years undergoing different academic streams, namely Medicine, Engineering, Dental, and Arts and Science, from various institutions in Chennai, South India, were invited to participate in the study, providing insights into headache prevalence and its triggers and impact. Among those included, those who were suffering from any neurological disorder or head and neck tumors were excluded from the study. The study was carried out cross-sectionally over two months, from September to October 2024.

Sampling and sample size

According to the study by Gururaj et al. [12], the one-year prevalence of headache (p) among adults was 63.9%, with a precision (d) of 4.5%, at a 95% confidence interval (Z₁₋ₐₗ₂ = 1.96). The sample size was calculated using the formula N = Z²₁₋ₐₗ₂ * p * (1 - p) / d² = 1.96² * 0.639 * (1 - 0.639) / 0.045² = 438. Snowball sampling was followed to reach as many young adults as possible in colleges of different streams to reach the sample size of 438.

Study tool and data collection

The study questionnaire was designed using questions from the Headache-Attributed Restriction, Disability, Social Handicap, and Impaired Participation (HARDSHIP) questionnaire to capture demographic details and diagnose headache types, triggers, and impact of headaches (see the Appendix). The HARDSHIP questionnaire, a structured questionnaire with a modular design, contains questions extracting data regarding demographic characteristics, screening for the presence of headache disorder, diagnosing headache type, and quantifying the burden and impact of headache disorder [13]. The questionnaire was validated in the Indian context [14]. Data collection was executed through a structured, digital approach. The questionnaire was then formatted in Google Forms and initially distributed to a carefully identified set of contacts within each of the target academic disciplines: Medicine, Dentistry, Engineering, and Arts and Science. A snowball sampling technique, a form of nonprobability sampling, was employed to extend the questionnaire's reach efficiently across these diverse fields. This approach allowed for leveraging participants' peer networks to foster the wide and rapid distribution of the survey link within each academic stream. Exclusion criteria were addressed through a preliminary section within the questionnaire, where subjects who self-reported a diagnosis of a neurological disorder or head and neck tumors were filtered out to maintain a focused sample population. Each initial participant was encouraged to share the survey with additional peers within their discipline, ensuring representation and depth of response across the academic streams while minimizing potential selection bias within the study. Data collection was conducted after getting approval from the Institutional Ethics Committee (IEC no: 309/09/2024/Faculty/SRB/SMCH).

Statistical analysis

The data collected were analyzed using Statistical Product and Service Solutions (SPSS) version 21 (IBM Corp., Armonk, NY). Categorical data were presented as frequencies and percentages, while continuous data were expressed as mean and standard deviation. A chi-square test was performed to test the association of the type of headache with impact, and an analysis of variance test was performed to test the significance of the difference between the mean duration of impact between headache types after checking for normality using the Shapiro-Wilk test. A p value less than 0.05 was considered significant.

## Results

The mean age of the participants was 20.1 years, ranging from 18 to 24 years. The population comprises 212 males (48.4%) and 226 females (51.6%). The participants are distributed almost equally across four academic disciplines: Medical (24.9%), Dental (25.1%), Engineering (25.1%), and Arts and Science (24.9%). A significant 60.9% of the participants (267 individuals) reported having experienced headaches in the past 12 months. Among them, 239 (54.6%) of them had headaches during the past 30 days. Among the 267 subjects with prevalence of headache, 121 (45.3%) were male and 146 (54.7%) were female participants. The average duration of the headache was around 4.6 days, ranging from 1 to 20 days (Table 1).

**Table 1:** Demographic characteristics and headache prevalence ^#^Expressed as mean (standard deviation) and range

Characteristics		Count (n = 438)	Percentage
Age (years)^#^		20.1 (0.94)	18-24
Gender	Male	212	48.4%
	Female	226	51.6%
Course of study	Medical	109	24.9%
	Dental	110	25.1%
	Engineering	110	25.1%
	Arts and Science	109	24.9%
Headache during the past 12 months	Yes	267	60.9%
	No	171	39.1%
Headache during the past 30 days	Yes	239	54.6%
	No	199	45.4%
Average duration of headache (days/month)^#^		4.6 (4.5)	1-20

For further analysis of study subjects regarding their headache, only the 267 subjects with the one-year prevalence of headaches were considered and studied. Over a quarter of the participants (27.3%) experience headaches that are mild or tolerable, and the majority report moderate (36.0%) to severe (36.8%) headaches. Self-management of headaches is common, with 43.9% using self-medication and 52.4% reporting symptom relief from medication. Only 17.9% seek professional help, despite 25.4% having a family history of headaches.

These data reflect a significant impact of headaches on daily life, with 73.0% experiencing activity limitations and 61.3% affected educationally. Social avoidance is reported by 36.5%, while 69.2% feel supported by family and friends. Substance use for relief is relatively low at 11.2%. On average, individuals report about 0.8 days of absenteeism, 1.7 days of reduced work productivity, and 1.8 missed leisure days over the past month due to headaches (Table 2).

**Table 2:** Characteristics and impact of headache ^#^Expressed as mean (standard deviation) and range

Characteristics	Count (n = 267)	Percentage
Severity of headache		
Not bad	73	27.3%
Quite bad	96	36.0%
Severely bad	98	36.8%
Duration of impact (days) due to headache^#^		
Absenteeism in the last 30 days	0.8 (2.0)	0-10
Perform less than half work in last 30 days	1.7 (3.2)	0-20
Missed leisure activities in last 30 days	1.8 (4.5)	0-10
Social impact		
Affects your day-to-day activities	195	73.0%
Interferes with your education	164	61.3%
Avoid people due to headache	97	36.5%
Acceptance from family and friends	185	69.2%
Substance abuse for relieving headache	30	11.2%
Treatment for headache		
Seek healthcare professional	48	17.9%
Headache relieved after medications	140	52.4%
Self-medications for headache	117	43.9%
Taking regular medications	53	19.9%
Family history of chronic headache disorder	68	25.4%

Personal stress is the primary headache trigger (38.7%), with academic stress (10.1%) and sinusitis (9.0%) also significant. Unknown causes account for 21.8%, indicating potential multifactorial origins. Physiological factors like sleeplessness, dehydration, and skipping meals are less frequent (Table 3).

**Table 3:** Triggers for headache

Triggers for headache	Count (n = 267)	Percentage
Do not know	80	21.8%
Sinusitis	33	9.0%
Academic stress	37	10.1%
Personal stress	142	38.7%
Sleeplessness	31	8.4%
Skipping meal	5	1.4%
Dehydration	6	1.6%
Watching TV	4	1.1%
Environmental stress	18	4.9%
Vision problem	7	1.9%
Mobile usage	4	1.1%

**Figure 1:**
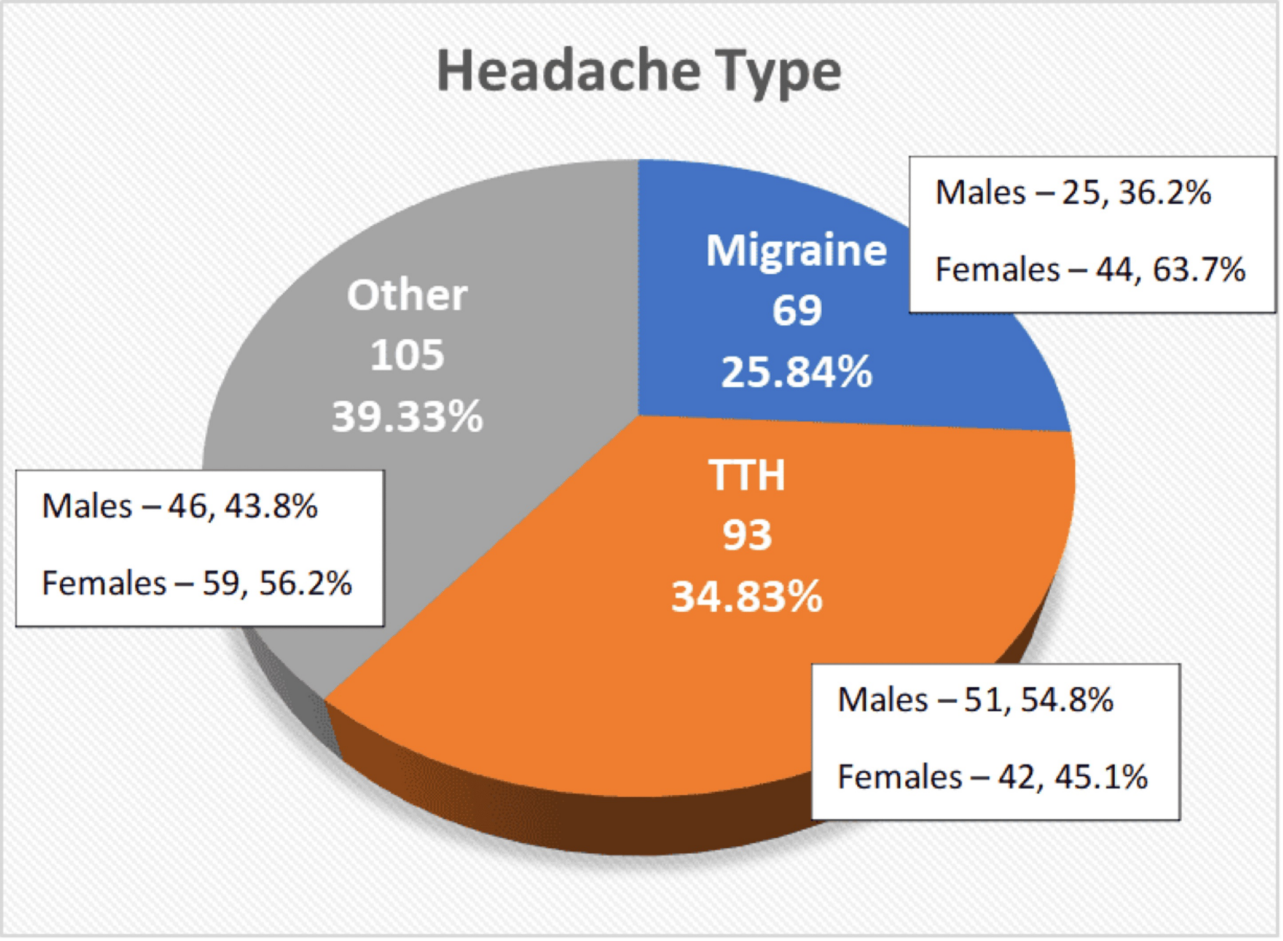
Diagnosis of headache type TTH: tension-type headache

The most common diagnosis is TTH, affecting over one-third (34.8%) of the population, and about a quarter (25.9%) of the population was affected by migraine. Almost 39% of the population had other types of headaches, which may be due to many reasons like lifestyle factors, physical exertion, neck issues, hormonal fluctuations, medication overuse, or trauma. Among those with migraine, female participants were very high (63.7%), while for those with TTH, male participants were slightly higher (54.8%) (Figure 1).

Individuals with migraines experience significantly more absenteeism (1.5 days on average) than those with TTH or other types, suggesting a higher impact on daily responsibilities for migraine sufferers. Migraines and TTH both result in notable reductions in work productivity of more than two days in the last month, but migraine sufferers experience a slightly higher impact. Leisure activities are most impacted for migraine sufferers, who miss an average of 3.1 days, followed by those with TTH (1.8 days) and other headaches (0.8 days), suggesting that migraines are significantly disruptive to personal life and recreational activities (Table 4).

**Table 4:** Duration of impact in different types of headaches The difference between the means were tested for significance using ANOVA test ANOVA: analysis of variance; TTH: tension-type headache

Duration of impact	Diagnosis			ANOVA F value	p value
	Migraine	TTH	Other		
Absenteeism in the last 30 days	1.5 (±2.7)	0.9 (±1.9)	0.4 (±1.3)	8.74	0.001
Perform less than half of the work in the last 30 days	2.3 (±3.5)	2.1 (±3.9)	0.9 (±2)	7.57	0.001
Missed leisure activities in the last 30 days	3.1 (±5.2)	1.8 (±5)	0.8 (±3.1)	7.97	0.001

A large majority of individuals with migraines (83.2%) and TTH (81.3%) report an impact on daily activities, while fewer with other headache types (59%) experience this. Migraine and TTH are particularly disruptive to daily routines compared to other headache types. Migraine sufferers experience the highest educational interference (83.2%), followed by those with TTH (64.1%) and other types (44.4%), indicating that migraines have the most severe impact on academic activities significantly. Those with migraines are more likely to avoid social interactions due to their headache (46.3%) compared to those with TTH (32.8%) and other headache types (33.3%). The difference was not significant. Across all headache types, the majority feel accepted and supported by family and friends, with no significant differences between the types of headaches. A family history of headache is more common among migraine sufferers (31.6%) than those with TTH (27.3%) or other headaches (19.4%), but there exists no statistically significant difference (Table 5).

**Table 5:** Social impact in different types of headaches The difference between the proportions was tested for significance using the chi-square test TTH: tension-type headache

Social impact	Diagnosis			χ^2^ value	p value
	Migraine	TTH	Other		
Affects your day-to-day activities	57 (83.2%)	76 (81.3%)	62 (59%)	17.21	0.001
Interferes with your education	57 (83.2%)	60 (64.1%)	47 (44.4%)	25.74	0.001
Avoids people due to headache	32 (46.3%)	31 (32.8%)	35 (33.3%)	3.74	0.144
Acceptance from family and friends	49 (71.6%)	61 (65.6%)	74 (70.8%)	0.741	0.646
Family history of headache	22 (31.6%)	25 (27.3%)	20 (19.4%)	3.89	0.169

## Discussion

This study aimed to assess the burden of headaches among young adults using the HARDSHIP questionnaire, revealing significant insights into headache prevalence, types, triggers, and their impact on daily functioning. The mean age of participants was 20.1 years, with a balanced gender distribution (48.4% male and 51.6% female participants). Notably, 60.9% of participants reported experiencing headaches in the past year, with 54.6% having headaches within the last 30 days. These findings highlight the considerable prevalence of headache disorders in this demographic, which is consistent with other studies conducted among young adults in India. Community and population-based surveys in Karnataka report a one-year prevalence of headache of 63.9% [12,15]. Menon and Kinnera found that approximately 68% of medical students in South India experienced headaches, indicating that headache disorders are a common issue among young adults [16]. A study by Nanda and Chhabra among dental students reported around 64% prevalence of headache disorders [17]. The prevalence of headaches among our participants (60.9%) aligns with findings from other studies in India that report similar rates among college students and young adults. The slight female predominance in headache reporting (54.7%) is consistent with Indian trends, suggesting that they are more likely to report migraines and TTHs [18,19]. This gender disparity may be attributed to hormonal influences and psychosocial factors that differentially affect women.

Our results indicate that while 27.3% of participants experience mild headaches that likely do not significantly affect their daily activities, a substantial proportion report moderate (36.0%) to severe (36.8%) headaches that can be disruptive or debilitating, often impacting productivity and requiring management. This finding resonates with the research by Kulkarni et al. [20], which reports 29% of mild headaches, 38% of moderate, and 32.5% of severe headaches. This highlights the significant burden of headache disorders on daily functioning and quality of life among young adults in India.

Self-management of headaches is prevalent in our population, with 43.9% using self-medication and only 17.9% seeking professional help despite a notable proportion (25.4%) having a family history of headaches. This low rate of professional help-seeking behavior is concerning and aligns with findings from other studies indicating barriers to accessing healthcare for headache management in India [21]. Headaches are frequently cited as a reason for self-medication, with prevalence rates of 62.04%-92.8% [22]. The reliance on self-medication may reflect a lack of awareness regarding effective treatment options or the stigma associated with seeking help for chronic pain conditions. With the 25% prevalence of a family history of headaches, the percentage seeking help remained low, indicating possible normalization of headache experiences within families or a belief in self-management efficacy.

It was found that 73% of the participants with headache disorders had their daily activities affected, and 61.3% had an impact on their education due to headaches. They reported about 0.8 days of absenteeism, 1.7 days of reduced work productivity, and 1.8 missed leisure days over the past month due to headaches. These findings reflect the substantial socioeconomic burden associated with headache disorders, as noted in studies emphasizing that headache-related disability can lead to decreased academic performance and increased absenteeism [7,9,10]. The findings from the study by Al-Hashel et al. [23] were similar to the current study and reported that absenteeism among students was around two days, and they could not perform usual activities for 2.84 days per month.

The prevalence of social avoidance (36.5%) in our study aligns with findings from research on migraine patients, which indicated that many individuals tend to cancel social activities during headache episodes [24]. This social withdrawal can exacerbate feelings of isolation and may contribute to a cycle of increased anxiety and headache frequency. Despite these challenges, most individuals across all headache types reported feeling accepted and supported by family and friends, suggesting that social support may play a protective role against the negative impacts of headaches on daily life.

Personal stress is the primary headache trigger (38.7%), followed by academic stress (10.1%), sinusitis (9.0%), and sleeplessness (8.4%). Stress can predispose individuals to headache disorders, accelerate their progression, and exacerbate individual episodes. The stress response involving the hypothalamic-pituitary-adrenal axis can lead to chronic activation that predisposes individuals to headaches [25]. Sleep disturbances are also a common trigger, with 41% of individuals reporting sleep-related headaches [26].

Among those with headache disorders, 35% were diagnosed with TTH, 26% with migraine, and the remaining 39% had other types of headache. Recent studies in India have revealed high prevalence rates of headache disorders among young adults. The study by Chowdhury et al. conducted in Delhi [27] reported a one-year prevalence of migraine at 26.3%, TTH at 34.1%, and other headaches at 7.5%. Similar findings were reported in Karnataka, with migraine at 25.2% and TTH at 35.1% [20]. The remaining 39% of participants diagnosed with other types of headaches reflect the complexity and diversity of headache disorders. Many individuals may experience mixed headache types or secondary headaches to other primary disorders affecting other organs systems such as cluster headaches or cervicogenic headaches, which can complicate diagnosis and management.

Migraine sufferers had higher levels of absenteeism and missing leisure activities, along with reduced work performance, compared to TTH and other headaches. This is supported by other research findings, which also show a loss of an average of two to three days due to migraine or TTH [28,29]. Migraines are associated with the highest levels of disruption in academic activities (83.2%) and daily routines (83.2%), a pattern consistent with their known pathophysiological features. Migraines are understood to involve complex neurovascular mechanisms, including abnormal excitability of the brain's cortex, particularly in response to sensory stimuli, leading to episodes of intense pain, photophobia, phonophobia, and other sensory disruptions [6]. The higher tendency of migraine sufferers to avoid social interactions (46.3%) also aligns with these sensory sensitivities, which can make social settings overwhelming.

Moreover, the data show that family history is slightly more common among migraine sufferers (31.6%) compared to TTH or other types, hinting at genetic factors involved in migraine pathophysiology. Studies indicate that a hereditary component in migraines may predispose individuals to neurotransmitter dysregulation, contributing to pain transmission and modulation [30]. In this light, the intense impact on educational and daily life may be partly due to both the physiological intensity and the often-intractable nature of migraine pain, which is linked to these neurobiological factors.

In comparison, TTH, while disruptive, generally involves less pronounced neurological sensitization and has been associated with muscular or myofascial pain pathways rather than the complex neurovascular mechanisms seen in migraines. This distinction could explain the lower but still significant impact of TTH on daily life and education.

The awareness about headache types, triggers, and effective management strategies, especially among young adults, should be increased, and this could be done by educational programs on college campuses highlighting the prevalence of headache disorders and promoting early intervention. Given the low rate of professional help-seeking behavior (17.9%) despite the high self-reported impact, interventions should target barriers to healthcare access for headaches. This may include subsidized treatment programs, confidential telehealth services, or on-campus headache clinics to facilitate accessible care. Public health campaigns on stress reduction, sleep hygiene, and regular healthcare checkups among young adults will definitely reduce the incidence of headaches and improve the social and vocational dimensions of the health of young adults.

The responses from the subjects were all self-reported, which can amount to recall bias to a certain extent, particularly for the frequency, severity, and impacts of headaches. Participants may underreport or overreport experiences based on their perceptions. While TTHs and migraines were well-categorized, less common headache types (e.g., cluster headaches and cervicogenic headaches) may be underrepresented or misclassified, limiting insights into these conditions. The study provided a snapshot of headache burden without tracking changes over time, such as seasonal variations, progression of headache disorders, or effects of intervention strategies. The study did not explore comorbid psychological or physical conditions (e.g., anxiety, depression, and neck or back pain), which are often associated with chronic headache disorders and could influence outcomes.

## Conclusions

This study emphasizes how common migraines and TTHs are highly prevalent among young adults, affecting both their everyday life and academic performance. Personal and academic stress and sleeplessness were the major triggers for headaches. The result show that in order to enhance headache treatment, decrease self-medication, and promote seeking professional assistance, there is a need for more awareness and focused interventions. In order to lessen the socioeconomic and psychological effects of headache disorders, educational initiatives, easily available medical care, and support networks might be extremely important. By tackling these issues, we can improve the impacted people's productivity and quality of life.

## Appendices

Questionnaire

The Burden of Headaches in Young Adults: Types, Triggers, and Impact on Daily Functioning Using Headache-Attributed Restriction, Disability, Social Handicap, and Impaired Participation (HARDSHIP) Questionnaire

Demographic details:

Name:                                                                                                          

Age: ______ years         

Gender: Male / Female

Course:

College:

Prevalence of headache:

1.      Have you had a headache during the past 12 months?

               ①   Yes                                ②   No

2.     Have you had a headache during the past 30 days?

               ①   Yes                                ②   No

3.  How often do you have this type of headache? _________ (average days/month)

4. How bad is this headache usually? ①   Not bad       ②   Quite bad             ③   Severely bad

Diagnosis of headache type:

5.  Is the pain of this type of headache usually on one side of the head?

               ①   Yes                                ②   No                              ③   NA

6.  Which of the following pain best describes your headache?

               ①   Throbbing/Pulsating           ②   Pressing/Squeezing/Tightening                   ③   NA 

7.  At what time of the day do you think your headache is at its maximum intensity?

               ①   Morning                    ②   Afternoon                 ③   Evening                      ④   NA

8.  With this type of headache, do you usually feel nauseated (sensation of vomiting)?

               ①   Yes                              ②   No                              ③   NA           

9.  Have you vomited during the headache?

               ①   Yes                              ②   No                              ③   NA    

10.  Do you have any associated giddiness or lightheadedness during your headache?

               ①   Yes                              ②   No                              ③   NA     

11.  Do you have any associated visual blurring during your headache?

               ①   Yes                              ②   No                              ③   NA       

12.  During this headache, does daylight or other lighting bother you?

               ①   Yes                              ②   No                              ③   NA 

13.  During this headache, does noise bother you?

               ①   Yes                              ②   No                              ③   NA

14.  Do you get recurrent nose block and runny nose often?

               ①   Yes                              ②   No                              ③   NA         

15.  Does this headache worsen with exercise like walking and climbing?

               ①   Yes                              ②   No                              ③   NA         

16.  Has a healthcare professional ever given you a diagnosis for this type of headache?

               ①   Yes                              ②   No                              ③   NA         

17.  If yes, what is the diagnosis? _____________________________________________

Impact of headaches:

18. Does this headache affect your ability to do day-to-day activities?

               ①   Can do everything as normal             ②   Can do something

               ③   Cannot do anything                              ④   NA

19.  Does your headaches interfere with your education/studies?

               ①   Yes                              ②   No                              ③   NA         

20. How many days in the last 30 days have you been unable to go to college because of your headache?          ________ Days

21. Not including the number of days you counted in the previous question, how many days in the last 30 days could you do only less than half of your usual college work because of headaches? ________ Days

22. How many days in the last 30 days did you miss family or leisure activities because of headaches?         ________ Days

23.  Do you avoid telling people that you have headaches?

               ①   Yes                              ②   No                              ③   NA         

24.  Do you feel that your family and friends understand and accept your headaches?

               ①   Yes                              ②   No                              ③   NA         

25.  Are you taking regular medications for your headache?

               ①   Yes                              ②   No                              ③   NA         

26.  Is your headache relieved after taking medications?

               ①   Yes                              ②   No                              ③   NA         

27.  Do you ever take self-medication for your headache?

               ①   Yes                              ②   No                              ③   NA         

28.  Do you have a family history of headaches?

               ①   Yes                              ②   No                              ③   NA         

29.  What do you believe is the reason or trigger for your headache? ______________________
